# Editorial: Combined EEG in research and diagnostics: Novel perspectives and improvements

**DOI:** 10.3389/fnins.2023.1152394

**Published:** 2023-02-16

**Authors:** Camillo Porcaro, Kamran Avanaki, Oscar Arias-Carrion, Morten Mørup

**Affiliations:** ^1^Department of Neuroscience and Padova Neuroscience Center, University of Padua, Padua, Italy; ^2^Institute of Cognitive Sciences and Technologies—National Research Council, Rome, Italy; ^3^Centre for Human Brain Health and School of Psychology, University of Birmingham, Birmingham, United Kingdom; ^4^University of Illinois at Chicago, Chicago, IL, United States; ^5^Unidad de Trastornos del Movimiento y Sueño, Hospital General Dr. Manuel Gea González, Mexico City, Mexico; ^6^Technical University of Denmark, Lyngby, Denmark

**Keywords:** EEG-fMRI, EEG-MEG, EEG-based BCI, EEG-TMS, EEG-tES, EEG-fNIRS

In neuroscience, electroencephalography and neuroimaging techniques are widely used to improve our understanding of brain mechanisms and to identify biomarkers for the most diverse neurological pathologies (Tulay et al., 2019). However, electro-magnetoencephalography (E-MEG) and neuroimaging techniques, such as functional magnetic resonance imaging (fMRI), are complementary [i.e., EEG/MEG techniques have an excellent temporal resolution at the expense of their spatial resolution and vice versa for fMRI or other neuroimaging techniques such as single-photon emission computed tomography (SPECT), positron emission tomography (PET) and functional near-infrared spectroscopy (fNIRS)]. Furthermore, the complementarity of these techniques has led to the development of multimodal integration (Tulay et al., 2019).

In recent decades, technological advances have allowed researchers to integrate different electrophysiological and neuroimaging techniques more efficiently to provide optimal spatial and temporal resolution. With its excellent spatial resolution and portability, EEG is often combined with other methods, such as fMRI (Ostwald et al., 2010, 2011, 2012; Porcaro et al., 2010, 2011) or fNIRS, transcranial magnetic stimulation (TMS) (Giambattistelli et al., 2014; Tecchio et al., 2023), and transcranial electrical stimulation (tES) (Porcaro et al., 2019b), to enhance the understanding of the brain functions underlying brain processes in healthy and pathological conditions (Buss et al., 2019). Moreover, EEG combined with non-invasive brain stimulation (NIBS) such as TMS, or tES can be used as a potential treatment and monitoring of brain pathologies (Napolitani et al., 2014; Cottone et al., 2018; Porcaro et al., 2019b). EEG, coupled with proper and advanced mathematical methods, can provide markers for neurodegenerative diseases and facilitate their diagnosis (Tecchio et al., 2015; Smits et al., 2016; Marino et al., 2019; Porcaro et al., 2019a, 2020, 2022a,b,c).

This Research Topic gives an overview of the current knowledge of EEG combined with other techniques for research and diagnostic purposes through 11 articles by 65 authors, which contain two reviews, eight original research papers and one method (Total views: 30,624; as of 27 Jan 2023).

One of the reviews focuses on investigating brain disorders using tES in combination with non-invasive neuroimaging techniques. The review highlights shortcomings and provides a comprehensive guideline for further investigation (Yang et al.). In particular, EEG and fNIRS were selected as noninvasive neuroimaging modalities in this systematic review. Nine brain disorders were investigated in this review, including Alzheimer's disease, depression, autism spectrum disorder, attention-deficit hyperactivity disorder, epilepsy, Parkinson's disease, stroke, schizophrenia, and traumatic brain injury. This review showed that most of the articles (82.6%) employed transcranial direct current stimulation (tDCS) as an intervention method with modulation parameters of 1 mA intensity (47.2%) for 16–20 min (69.0%) duration of stimulation in a single session (36.8%). The author concluded that future work needs to investigate a closed-loop tES with monitoring by neuroimaging techniques to achieve personalized therapy for brain disorders.

The second review includes a flow chart of questions that researchers can consider when deciding whether to record EEG and fMRI separately or simultaneously (Scrivener). Overall, this article aims to equip new researchers with the resources needed to make an informed decision regarding the necessity of simultaneous EEG-fMRI. As multi-modal neuroimaging requires additional time, equipment, and financial resources, it is essential to consider the recording options available thoroughly. Furthermore, ongoing technological and methodological developments continue to facilitate the successful application of combined EEG-fMRI to answer questions about the brain and behavior with increasing precision.

In addition to the reviews, eight original studies analyze the combination of electrophysiological techniques. While some studies focused on combining EEG and Electromyography (EMG) others combined electro- and magnetoencephalography (E-MEG). One of the studies combining EEG and EMG (Zhao et al.) showed robust relationships between EEG and EMG signals that might be of some interest for analyzing neuromotor disorders, such as Parkinson's disease, to identify neural correlates of abnormal gait. In another study, the authors (Pascarella et al.) have developed a new measure named normalized compression distance (NCD) to measure cortico-muscular synchronization using EEG and EMG data. A third study employed a coupled tensor decomposition to extract the signal sources from MEG-EEG during intermittent photic stimulation (IPS). There, Coupled Semi-Algebraic framework for approximate CP decomposition *via* SImultaneous matrix diagonalisation (C-SECSI) was able to separate physiologically meaningful oscillations of visually evoked brain activity from background signals. The component frequencies are able to identify either an entrainment to the respective visual stimulation frequency, its first harmonic, or an oscillation in the individual alpha band or theta band. A reciprocal relationship between alpha and theta band oscillations is present in the group analysis of both EEG and MEG data. The coupled tensor decomposition using the C-SECSI framework is a robust, powerful method for the unsupervised extraction and separation of meaningful sources from multidimensional biomedical measurement data (Naskovska et al.). Finally, another study combined EEG and MEG data using microstates modeled from subject- and modality-specific archetypes that represent distinct topographic maps between which the brain continuously traverses. The implemented method successfully models scale and polarity invariant data, such as microstates, accounting for intersubject and intermodal variability. Furthermore, the model is readily extendable to other modalities ensuring component correspondence while elucidating spatiotemporal signal variability (Olsen et al.).

The utility of electroencephalography for diagnostics is further highlighted both considering Alzheimer's disease (AD) and Stroke. For AD, topological features of networks constructed for each EEG channel based on weighted visibility graphs were considered in Yu et al. and deep learning computer vision models were applied to image representations of topographic and spectral properties of the EEG in Jeong et al.. For stroke, the properties of EEG microstates, including changes in functional connectivity patterns were explored in Hao et al.. Additionally, the use of EEG for the quantification of treatment effect is investigated in the context of remote ischemic preconditioning (RIPC) in Li et al., whereas a modeling framework based on fNIRS as a complementary approach to EEG is proposed for the discrimination of single trial task responses for brain computer interfaces (BCI) in Zhang et al..

Overall, this Research Topic shows the fronts in combining EEG with other techniques to study dynamic brain functions or changes from a temporal and spatial perspective. These include technical possible study design, data acquisition, and data analysis to improve human health by combining advanced brain technologies with cutting-edge science and original bio-medical insight. This challenge requires creative problem-solving, precision and a lot of imagination. There is a big challenge in removing artifacts to perform fully continuous EEG-fMRI/TMS/tDCS/tES recordings. This will be the mainstay of multimodal functional brain imaging. Neuroimaging-techniques is an area that needs further research and validation of algorithms.

Finally, clinical applications have thus far been limited. In the future, combined neuroimaging studies will help neuroscientists to extract neuro-information underlying sensory and cognitive activity in healthy and pathological conditions.

## Author contributions

All authors contributed to the article and approved the submitted version.
